# Exploring dietary habits strongly associated with pancreatic cancer from the perspective of Mendelian randomization

**DOI:** 10.1097/MD.0000000000047721

**Published:** 2026-02-20

**Authors:** Sujuan Chen, Yuchao Liu, Ting Fan, Haiting Yuan, Tao Guo, Junchao Wang

**Affiliations:** a Department of Oncology, Foguang Hospital of Emei Mountain, Emeishan, Sichuan, China; b Department of Oncology, The Armed Police Forces Hospital of Sichuan, Leshan, Sichuan, China; c Department of Hepatobiliary Surgery, Foguang Hospital of Emei Mountain, Emeishan, Sichuan, China; d Department of Radiation Oncology, Sichuan Cancer Hospital & Institute, Sichuan Cancer Center, Affiliated Cancer Hospital of University of Electronic Science and Technology of China, Chengdu, Sichuan, China.

**Keywords:** alcohol consumption, dietary habits, genetic epidemiology, Mendelian randomization, pancreatic cancer, UK biobank, vegetable intake

## Abstract

While some retrospective studies have reported that certain dietary habits may affect pancreatic cancer, the variety of dietary habits studied has been limited. A 2-sample Mendelian randomization (MR) analysis was performed using exposure data from the UK Biobank data (n = 10,019,305) and outcome data from the FinnGen database (731 pancreatic cancer cases and 314,193 controls). Instrumental variables were selected based on genome-wide significance, followed by linkage disequilibrium clumping. Sensitivity analyses, including Steiger filtering and MR-PRESSO, were conducted to ensure the robustness of the causal inferences. Following this process, we extracted and filtered the data, ultimately selecting 30 dietary phenotypes from the original 231. Our findings revealed that alcohol intake frequency (raw *P*-value =.002, odds ratio (OR) = 1.090, 95% CI: 1.032–1.151) and average weekly spirits intake (raw *P*-value = .005, OR = 1.793, 95% CI: 1.196–2.688) were risk factors for pancreatic cancer using data from 706 single nucleotide polymorphisms derived from FinnGen database. Notably, cooked vegetable intake (raw *P*-value = .029, OR = 0.704, 95% CI: 0.514–0.965) was found to have a protective effect against pancreatic cancer. Our MR analysis provides evidence that specific dietary habits significantly influence pancreatic cancer risk, highlighting the potential of dietary interventions within public health strategies for pancreatic cancer management.

## 1. Introduction

Pancreatic cancer is a highly lethal malignancy, primarily characterized by the development of pancreatic ductal adenocarcinoma, which constitutes approximately 90% of cases. This aggressive neoplasm is often asymptomatic in its early stages, leading to late diagnosis and a dismal prognosis, with a 5-year survival rate of approximately 13%.^[1]^ The clinical classification of pancreatic cancer includes subtypes such as ductal adenocarcinoma, adenosquamous carcinoma, and mucinous cystadenocarcinoma, each with varying survival outcomes.^[2]^ Major etiological factors for pancreatic cancer include genetic mutations, particularly in the KRAS, CDKN2A, TP53, BRCA1/2 and ATM genes, as well as environmental factors such as smoking, obesity, and chronic pancreatitis.^[3]^ The pathophysiological process involves a sequence of genetic mutations leading to the malignant transformation of pancreatic epithelial cells, progressing through stages of pancreatic intraepithelial neoplasia to invasive carcinoma.^[4]^ Epidemiologically, pancreatic cancer is the sixth leading cause of cancer-related deaths globally, with a rising incidence due to factors like aging populations and increasing prevalence of risk factors such as diabetes and obesity.^[5]^ The economic burden of pancreatic cancer is significant, as it often requires intensive treatment and prolonged hospital stays, contributing to high healthcare costs and substantial loss of productivity.^[1]^ Given the severe impact of pancreatic cancer on both individual health and public health systems, ongoing research is crucial to identify more effective screening methods, therapeutic targets, and preventive strategies to reduce the morbidity and mortality associated with this disease.

Several studies have explored the relationship between dietary habits and pancreatic cancer, revealing a significant association between unhealthy dietary patterns, such as high-fat diets, and the incidence of pancreatic cancer. Research utilizing murine models has demonstrated that high-fat diets not only increase tumor volume but also enhance tumor angiogenesis and reduce apoptosis, resulting in a more aggressive pancreatic cancer phenotype.^[6]^ Furthermore, large-scale prospective cohort studies have demonstrated a strong association between metabolic syndrome and an increased risk of pancreatic cancer.^[7]^ However, a notable limitation of these studies is their lack of a thorough analysis of the complex relationship between dietary composition and metabolic biomarkers. Research on nutritional factors related to pancreatic cancer has predominantly focused on obesity and its associated metabolic disturbances. Obesity-induced adipose tissue inflammation is considered a key factor in the promotion of pancreatic cancer, with particularly strong associations found between intrapancreatic fat deposition and pancreatic cancer growth and progression.^[8]^ A systematic review and meta-analysis further confirmed the relationship between obesity and components of metabolic syndrome, such as hyperglycemia and low high-density lipoprotein cholesterol, with a significantly increased risk of pancreatic cancer. Nonetheless, the heterogeneity of these findings and inadequate control for other potential confounders remain challenges.^[9]^

Many studies are constrained by observational designs, which are prone to confounding and reverse causation, thereby weakening the ability to infer causality between diet and cancer risk. Additionally, retrospective studies and randomized controlled trials often face issues such as recall bias, limited sample sizes, and ethical constraints, which can undermine the robustness of their findings. Mendelian randomization (MR) offers a robust alternative by utilizing genetic variants as instrumental variables (IVs) to assess causal effects, minimizing biases inherent in observational studies.^[10]^ While recent MR studies have examined the role of metabolic traits like obesity^[11]^ and type 2 diabetes^[12]^ in pancreatic cancer, the causal relationship between specific, modifiable dietary habits and pancreatic cancer risk remains largely unexplored using this methodology.

Therefore, we hypothesize that specific dietary habits (e.g., frequent alcohol consumption, low vegetable intake) have a causal effect on the risk of developing pancreatic cancer. To test this hypothesis, we employed a 2-sample MR analysis to investigate the causal effects of a wide range of dietary phenotypes on pancreatic cancer incidence, leveraging large-scale genetic data from the UK Biobank and FinnGen consortium.

## 2. Methods and materials

### 2.1. Study design and data sources

This study employed a 2-sample MR analysis, with dietary habits as the exposure variable and pancreatic cancer as the outcome, to explore the causal relationship between these 2 factors using statistical methodologies.^[13]^ The main results are listed in Supplementary Material S1, Supplemental Digital Content, https://links.lww.com/MD/R415. In MR studies, single nucleotide polymorphisms (SNPs) are used as IVs due to their inherent randomness in genetic inheritance and their presence from conception, thereby providing temporal extension and minimizing confounding factors and reverse causation bias. The details are listed in Supplementary Material S2, Supplemental Digital Content, https://links.lww.com/MD/R414. The validity of MR results hinges on 3 key principles: a strong association between the IVs and the exposure, the absence of a significant correlation between the IVs and confounders, and the inability of the IVs to directly affect the outcome.^[13,14]^ When these principles are upheld, the MR results are considered robust.

The data for this study were divided into 2 main categories: exposure phenotypes (dietary habits) and outcomes (pancreatic cancer). After a rigorous selection process, the data underwent subsequent statistical analysis. The exposure data were sourced from Ben Elsworth and colleagues, who processed the UK Biobank data via the MRC-IEU. The initial dataset included 10,019,305 samples from a European population, representing 231 dietary habits, which were narrowed down to 30 phenotypes after stringent selection criteria. The outcome data were obtained from the FinnGen database, comprising 731 case samples and 314,193 control samples of European descent related to pancreatic cancer. These datasets are publicly available through the GWAS Catalog (GWAS Catalog [ebi.ac.uk]) or the FinnGen database (https://storage.googleapis.com/finngen-public-data-r10/summary_stats/finngen_R10_C3_PANCREAS_ADENO_DUCTAL_EXALLC.gz), with detailed descriptions provided in Supplementary Material S3, Supplemental Digital Content, https://links.lww.com/MD/R415.

### 2.2. Selection of genetic instrument variables

To ensure the reliability of the MR analysis, the selection and extraction of IVs were guided by 3 fundamental principles. First, to ensure that the IVs adequately represent the exposure phenotypes, we set a correlation threshold of *P* < 5 × 10^−8^ between the IVs and the exposure.^[15]^ Second, we excluded confounders such as smoking and type 2 diabetes from the GWAS Catalog database to reduce potential confounding effects. To minimize reverse causality in the MR analysis, we employed the Steiger test,^[16]^ retaining data with a *P*-value <.05 and discarding data more strongly related to the outcome. Additionally, to ensure the independence of SNPs and avoid linkage disequilibrium, we set a window size of 10,000 kb and *R*^2^ <0.01. We also set an *F*-value threshold of 10 to mitigate the impact of weak instruments. To minimize potential bias caused by ambiguous alleles or palindromic SNPs during the process of aligning effect alleles between exposure and outcome datasets, we implemented the following quality control steps: first, we excluded SNPs with a minor allele frequency below 0.01, as such variants are more prone to misclassification or strand ambiguity and may introduce bias in effect allele harmonization. Second, in addition to the genome-wide significance threshold (*P* < 5 × 10^−^8) used for primary IV selection, we applied an additional statistical threshold of *P* < 5 × 10^−2^ for secondary validation steps in certain sensitivity analyses, ensuring that only robust and reliably measured SNPs were retained for the final MR estimates.

### 2.3. Mendelian randomization analysis

This study utilized 5 MR methods to evaluate the causal effect between exposure and outcome: inverse-variance weighted (IVW),^[17]^ Weighted Median,^[18]^ MR-Egger,^[19]^ Weighted Mode, and Simple Mode. IVW served as the primary method, as it requires the absence of invalid SNPs and horizontal pleiotropy for the most robust results. In contrast, Weighted Median allows up to 50% of SNPs to be invalid or exhibit horizontal pleiotropy, while MR-Egger, which permits all SNPs to be invalid, provides a more conservative approach and complements IVW. The Weighted Mode and Simple Mode methods were used as additional controls. When the causal effect directions of IVW, Weighted Median, and MR-Egger are consistent (statistically expressed as ORs either all > or <1), the results are considered robust. If all 5 MR methods yield consistent results, the findings are deemed highly robust. Moreover, an OR value >1 indicates a pathogenic causal effect, while an OR <1 suggests a protective causal effect. A 95% confidence interval that crosses 1 typically indicates that the results are not statistically significant.

### 2.4. Quality control

To ensure the rigor of the study results, we implemented a series of stringent quality control measures, organized into 3 levels: sensitivity analysis, heterogeneity analysis, and reverse causality analysis. For sensitivity analysis, we primarily employed Egger-intercept, MR-PRESSO, and leave-one-out (LOO) methods. The Egger-intercept, expressed as the intercept value of a linear regression, is significant when the *P*-value is <.05, indicating potential horizontal pleiotropy when the exposure factor is plotted on the x-axis and the outcome factor on the y-axis, and the outcome exists at the origin. MR-PRESSO is a more sensitive method,^[20]^ capable of detecting global horizontal pleiotropy; a *P*-value <.05 often indicates its presence. The LOO method^[21]^ systematically removes SNPs one at a time to observe their impact on the overall causal effect, identifying outliers, with results typically displayed in a forest plot.

Heterogeneity analysis assesses the compatibility of individual SNPs.^[20]^ When heterogeneity is present, the combined overall effect is generally unreliable. We conducted Cochran *Q* test using MR-IVW and MR-Egger regression methods and considered heterogeneity significant when *P* <.05. Lastly, reverse causality was analyzed using both the Steiger global reverse causality test and reverse MR analysis. When the Steiger test’s *P*-value is <.05, the risk of reverse bias is considered low. In reverse MR, we swapped the roles of exposure and outcome – in this study, treating pancreatic cancer as the exposure and dietary habits as the outcome for causal effect analysis. Importantly, the same standards for instrument variable selection and experimental methods applied, except that dietary habits could not be reused as IVs to avoid violating the 3 key MR principles. A *P*-value >.05 in the reverse MR indicates no statistical significance, suggesting a low risk of reverse bias.

### 2.5. Statistical analysis

The MR analysis in this study was conducted using statistical techniques, primarily relying on R version 4.3.1, alongside TwoSampleMR^[22]^ and MR-PRESSO packages for analysis and quality control. We set the significance threshold at *P* <.05 and calculated OR values and 95% confidence intervals for the 5 MR methods. An OR >1 was considered indicative of a pathogenic causal effect, while an OR <1 suggested a protective causal effect. Quality control measures were conducted by calculating relevant *P*-values and associated metrics, and only results meeting the specified criteria were considered positive.

## 3. Results

### 3.1. Genetic IVs selection

**Figure 1.**
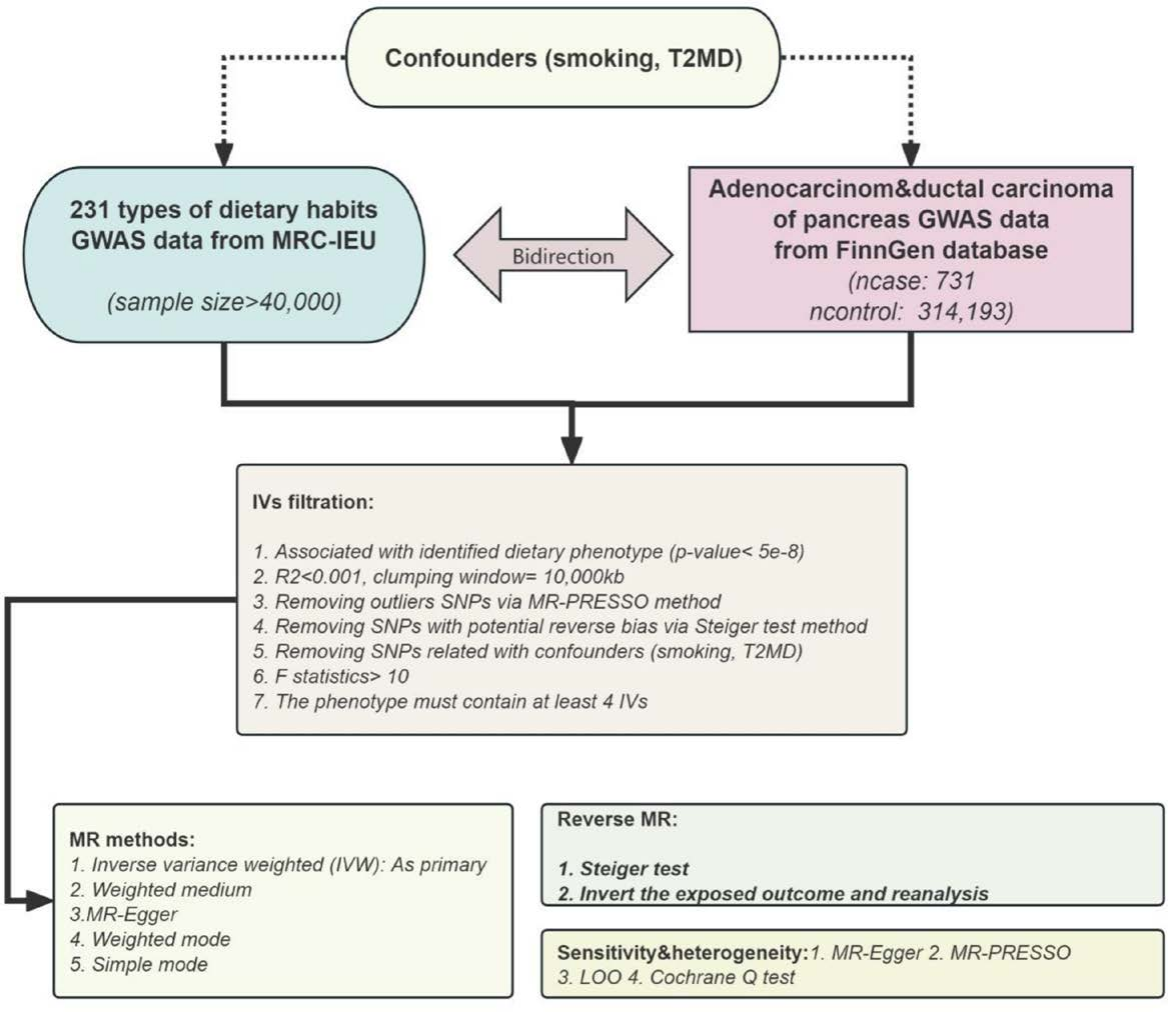
Diagram of work design and flow.

Based on the described process, we extracted and filtered the data, ultimately selecting 30 dietary phenotypes from the original 231 dietary habit phenotypes. These phenotypes included: alcohol intake frequency, average weekly spirits intake, cooked vegetable intake, fresh fruit intake, sushi intake, dried fruit intake, unsalted peanuts intake, pancake intake, processed meat intake, snack pot intake, pork intake, bread intake, Scotch egg intake, tea intake, poultry intake, average weekly beer plus cider intake, salad/raw vegetable intake, water intake, coffee intake, green tea intake, alcohol intake compared to 10 years prior, average weekly red wine intake, lamb/mutton intake, nonoily fish intake, beef intake, herbal tea intake, tofu intake, cereal intake, oily fish intake, and cheese intake. Among these, alcohol intake frequency, average weekly spirits intake, and cooked vegetable intake showed positive associations. The research process is detailed in Figure 1.

We then proceeded to extract the SNPs associated with these phenotypes according to the previously mentioned IVs extraction method. After controlling for multiple confounding factors, a total of 706 SNPs were selected for further analysis. Detailed data can be found in Table 1.

**Table 1:** Phenotypic information on dietary habits.

id	Trait	Population	Sex	Unit	Year	Sample size	NSNP	Consortium	Author
ukb-b-5779	Alcohol intake frequency.	European	Males and females	SD	2018	462,346	9,851,867	MRC-IEU	Ben Elsworth
ukb-b-1707	Average weekly spirits intake	European	Males and females	SD	2018	326,565	9,851,867	MRC-IEU	Ben Elsworth
ukb-b-8089	Cooked vegetable intake	European	Males and females	SD	2018	448,651	9,851,867	MRC-IEU	Ben Elsworth
ukb-b-3881	Fresh fruit intake	European	Males and females	SD	2018	446,462	9,851,867	MRC-IEU	Ben Elsworth
ukb-b-5213	Sushi intake	European	Males and females	SD	2018	64,949	9,851,867	MRC-IEU	Ben Elsworth
ukb-b-16576	Dried fruit intake	European	Males and females	SD	2018	421,764	9,851,867	MRC-IEU	Ben Elsworth
ukb-b-15555	Unsalted peanuts intake	European	Males and females	SD	2018	64,949	9,851,867	MRC-IEU	Ben Elsworth
ukb-b-6500	Pancake intake	European	Males and females	SD	2018	64,949	9,851,867	MRC-IEU	Ben Elsworth
ukb-b-6324	Processed meat intake	European	Males and females	SD	2018	461,981	9,851,867	MRC-IEU	Ben Elsworth
ukb-b-12912	Snackpot intake	European	Males and females	SD	2018	64,946	9,851,867	MRC-IEU	Ben Elsworth
ukb-b-5640	Pork intake	European	Males and females	SD	2018	460,162	9,851,867	MRC-IEU	Ben Elsworth
ukb-b-11348	Bread intake	European	Males and females	SD	2018	452,236	9,851,867	MRC-IEU	Ben Elsworth
ukb-b-13516	Scotch egg intake	European	Males and females	SD	2018	64,949	9,851,867	MRC-IEU	Ben Elsworth
ukb-b-6066	Tea intake	European	Males and females	SD	2018	447,485	9,851,867	MRC-IEU	Ben Elsworth
ukb-b-8006	Poultry intake	European	Males and females	SD	2018	461,900	9,851,867	MRC-IEU	Ben Elsworth
ukb-b-5174	Average weekly beer plus cider intake	European	Males and females	SD	2018	327,634	9,851,867	MRC-IEU	Ben Elsworth
ukb-b-1996	Salad/ raw vegetable intake	European	Males and females	SD	2018	435,435	9,851,867	MRC-IEU	Ben Elsworth
ukb-b-14898	Water intake	European	Males and females	SD	2018	427,588	9,851,867	MRC-IEU	Ben Elsworth
ukb-b-5237	Coffee intake	European	Males and females	SD	2018	428,860	9,851,867	MRC-IEU	Ben Elsworth
ukb-b-4078	Green tea intake	European	Males and females	SD	2018	64,949	9,851,867	MRC-IEU	Ben Elsworth
ukb-b-3460	Alcohol intake versus 10 years previously	European	Males and females	SD	2018	428,117	9,851,867	MRC-IEU	Ben Elsworth
ukb-b-5239	Average weekly red wine intake	European	Males and females	SD	2018	327,026	9,851,867	MRC-IEU	Ben Elsworth
ukb-b-14179	Lamb/mutton intake	European	Males and females	SD	2018	460,006	9,851,867	MRC-IEU	Ben Elsworth
ukb-b-17627	Nonoily fish intake	European	Males and females	SD	2018	460,880	9,851,867	MRC-IEU	Ben Elsworth
ukb-b-2862	Beef intake	European	Males and females	SD	2018	461,053	9,851,867	MRC-IEU	Ben Elsworth
ukb-b-13344	Herbal tea intake	European	Males and females	SD	2018	64,949	9,851,867	MRC-IEU	Ben Elsworth
ukb-b-5522	Tofu intake	European	Males and females	SD	2018	64,945	9,851,867	MRC-IEU	Ben Elsworth
ukb-b-15926	Cereal intake	European	Males and females	SD	2018	441,640	9,851,867	MRC-IEU	Ben Elsworth
ukb-b-2209	Oily fish intake	European	Males and females	SD	2018	460,443	9,851,867	MRC-IEU	Ben Elsworth
ukb-b-1489	Cheese intake	European	Males and females	SD	2018	451,486	9,851,867	MRC-IEU	Ben Elsworth

### 3.2. Main MR results

We conducted 5 types of MR analyses following the methodology outlined above, with the IVW method serving as the primary analysis and the others as supplementary. A *P*-value of <.05 in the IVW analysis was considered statistically significant. For each analysis, we calculated the odds ratio (OR); OR values >1 were interpreted as indicative of a pathogenic causal effect, while OR values <1 were interpreted as indicative of a protective causal effect. Ultimately, we identified 3 significant associations: alcohol intake frequency, average weekly spirits intake, and cooked vegetable intake.

We report these significant findings in 2 categories. First, the pathogenic results: alcohol intake frequency (raw *P*-value = .002, OR = 1.090, 95% CI: 1.032–1.151) and average weekly spirits intake (raw *P*-value = .005, OR = 1.793, 95% CI: 1.196–2.688). Both phenotypes consistently demonstrated a significant pathogenic causal effect across all 5 MR methods, with OR values >1. This consistency suggests that these dietary habits are robustly associated with an increased risk of pancreatic cancer.

Second, we highlight the protective result: cooked vegetable intake (raw *P*-value = .029, OR = 0.704, 95% CI: 0.514–0.965). This phenotype also showed a consistent and significant protective causal effect across all 5 MR methods, with OR values <1, indicating a strong inverse association with the development of pancreatic cancer.

**Figure 2.**
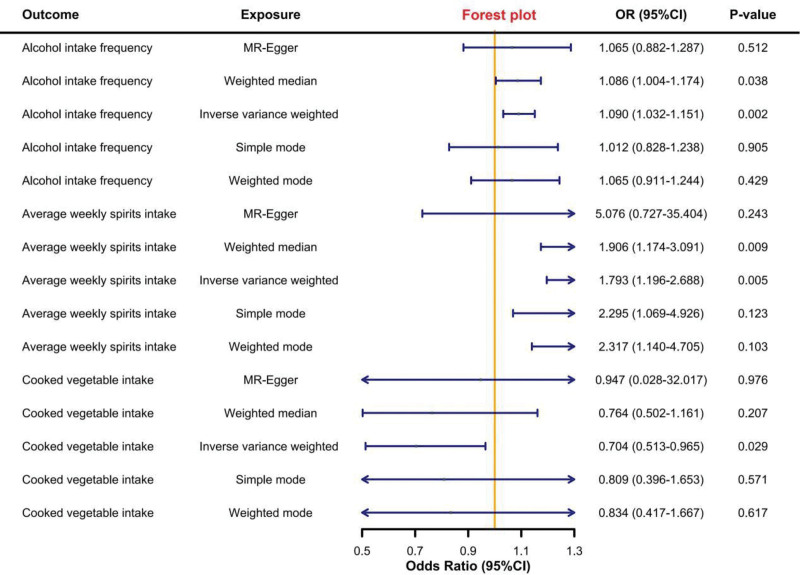
The forest map display of the main results. The above results are positive results with a *P*-value <.05 and no significant pleiotropy after FDR correction. FDR = false discovery rate.

The main results of the 5 MR analyses are presented in Figure 2, and the detailed data for all phenotypes are available in Supplementary Material S1, Supplemental Digital Content, https://links.lww.com/MD/R415.

### 3.3. Sensitivity and heterogeneity

To ensure the reliability of our results, we implemented rigorous quality control following the aforementioned methodology. In the sensitivity analysis, we utilized the Egger-intercept, MR-PRESSO, and LOO methods to evaluate the 3 significant phenotypes. None of these tests indicated the presence of outliers, suggesting that there was no significant pleiotropy or influential outlier effects.

Regarding heterogeneity analysis, we performed Cochran Q test on the MR-IVW and MR-Egger results for the 3 significant phenotypes, and no outliers were found, indicating the absence of heterogeneity among the results. In terms of reverse causation, the *P*-values from the Steiger test for the 3 significant phenotypes were all substantially below .05, and the reverse MR analyses yielded no statistically significant results. Therefore, we conclude that the risk of reverse confounding is minimal.

## 4. Discussion

This study, analyzing 231 dietary phenotypes, reveals a positive correlation between the frequency of alcohol consumption and the average weekly intake of spirits with the incidence and progression of pancreatic cancer. In contrast, the intake of cooked vegetables demonstrates a negative correlation with the development of pancreatic cancer. These findings could provide valuable guidance for dietary health in patients with pancreatic diseases and highlight the importance for clinical practitioners to conduct timely cancer prescreening for patients exhibiting dietary habits positively associated with this type of cancer. Such proactive measures may help prevent the progression of the disease toward more severe outcomes. Dietary components are believed to play a significant role in the development of this disease; risk factors include obesity, high consumption of red meat, and fried foods.^[23,24]^ Conversely, several studies suggest that diets rich in vegetables, fresh fruits, nuts, and whole grains may contribute to the prevention of pancreatic cancer.^[25]^ Our research suggests a causal link between alcohol intake, the consumption of cooked vegetables, and the incidence of pancreatic cancer, thus may offer dietary guidance for future patients and provide empirical support for further research in this domain.

Heavy alcohol consumption increases pancreatic cancer.^[26]^ In contrast, a meta-analysis indicated that both light and moderate alcohol consumption did not correlate with PC risk, while a dose-response relationship existed between higher alcohol intake and increased risk.^[27]^ Research conducted by Chang et al identified a strong association between heavy drinking and elevated PC risk, suggesting that the accumulation of acetaldehyde, a byproduct of human alcohol metabolism, may accelerate tumor progression by promoting pancreatic inflammation.^[28]^ Furthermore, genetic polymorphisms in the ALDH2 enzyme among East Asian populations, particularly the presence of the ALDH2 × 2 allele, have been found to heighten the risk of alcohol-related cancers.^[29,30]^

In addition, a recent systematic review showed that alcohol consumption has become a potential nongenetic risk factor for pancreatic ductal adenocarcinoma.^[31]^ The principal carcinogenic mechanism of alcohol is linked to its metabolite acetaldehyde, identified as a carcinogen in various in vitro, human, and animal studies.^[32]^ Chronic alcohol-induced pancreatitis, stemming from prolonged excessive drinking, may partially explain the association between alcohol consumption and pancreatic cancer risk.^[33]^ Our findings demonstrate a positive correlation between alcohol intake frequency (raw *P* = .002; OR = 1.090; 95% CI: 1.032–1.151) and average weekly spirits intake (raw *P* = .005; OR = 1.793; 95% CI: 1.196–2.688) with the incidence and progression of pancreatic cancer. These results align with current research, adding further empirical evidence to this field of study.

A study suggests that a diet high in vegetables, whole grains, and nuts is associated with lower cancer incidence and mortality and may favorably alter the gut microbiome.^[34]^ The consumption of a diverse range of fruits and vegetables, particularly leafy greens, cruciferous vegetables, berries, and citrus fruits, is associated with a reduced risk of developing pancreatic cancer.^[35]^ The mechanisms underlying the anticancer properties of these phytochemicals include antioxidant and anti-inflammatory effects; inhibition of cell proliferation, differentiation, adhesion, and invasion; as well as antimicrobial and antiviral actions that stimulate immune function; DNA damage repair; regulation of steroid hormones and estrogen metabolism; modulation of signaling pathways; enzyme regulation; suppression of oncogene expression while inducing tumor suppressor gene expression; activation of cell cycle G1 arrest; and induction of cellular differentiation and apoptosis.^[36]^ Our research indicates that cooked vegetable intake is associated with a protective causal effect against pancreatic cancer (raw *P* = .029; OR = 0.704; 95% CI: 0.514–0.965), supporting the existing notion of a negative correlation between vegetable consumption and the incidence and progression of pancreatic cancer. This finding offers guidance for health-conscious dietary choices for patients with pancreatic-related diseases.

This study utilized MR analysis, employing genetic variables (SNPs) as IVs, which introduces a level of randomization at the genetic level. This approach mitigates confounding factors and reduces the likelihood of reverse causation. The data source from the UK Biobank and the FinnGen database is extensive, providing a large sample size that enhances the reliability of the findings.^[37]^ Moreover, various quality control measures were implemented, contributing to the robustness of the results. This study has several limitations. First, the number of pancreatic cancer cases (n = 731) in the FinnGen outcome dataset, while substantial, is relatively low for a genetic study and may limit the statistical power to detect more subtle associations. This also necessitates caution in generalizing the findings, and replication in larger consortia is warranted. Second, our study is primarily based on European populations, which limits its applicability to other demographic groups. Third, stringent selection criteria resulted in the loss of certain phenotypes. Fourth, the study did not provide detailed stratification by age and gender, which could affect the granularity of the findings. To enhance the credibility of the results, external validation from additional databases and analyses in diverse populations are needed in the future.

## 5. Conclusion

Our findings highlight the significant role of specific dietary habits in the risk of pancreatic cancer. The results could inform dietary intervention strategies in public health policy.

## Author contributions

**Data curation:** Yuchao Liu.

**Formal analysis:** Haiting Yuan, Tao Guo.

**Software:** Ting Fan.

**Writing – original draft:** Sujuan Chen.

**Writing – review & editing:** Junchao Wang.

## Supplementary Material
